# Relationship between *p53* gene codon-72 polymorphisms and hypertrophic scar formation following caesarean section

**DOI:** 10.3892/etm.2014.1605

**Published:** 2014-03-05

**Authors:** JIANHUA GAO, YING CHEN, NONG LIAO, WEI ZHAO, WEISEN ZENG, YINGTAO LI, SHAOJING WANG, FENG LU

**Affiliations:** 1 Department of Plastic and Reconstructive Surgery, Nanfang Hospital, Southern Medical University, Guangzhou, Guangdong 510515, P.R. China; 2 Department of Breast Surgery, Guandong Traditional Chinese Medicine Hospital, Guangzhou, Guangdong 510000, P.R. China; 3 Department of Plastic Surgery, The Third Affiliated Hospital of Guangzhou Medical College, Guangzhou, Guangdong 510150, P.R. China; 4 Department of Cell Biology, Southern Medical University, Guangzhou, Guangdong 510515, P.R. China; 5 Department of Obstetrics and Gynecology, The Third Affiliated Hospital of Guangzhou Medical College, Guangzhou, Guangdong 510150, P.R. China

**Keywords:** *p53* gene codon-72, hypertrophic scar, molecular beacon quantitative polymerase chain reaction

## Abstract

The aim of the present study was to determine the relationship between *p53* gene codon-72 polymorphisms and hypertrophic scar formation following caesarean section (CS). Blood samples from 260 female patients were collected one week following a CS for the detection of *p53* gene polymorphisms using a molecular beacon-coupled quantitative polymerase chain reaction technique. Patients had follow-ups for 12–18 months to observe the scar formation. From these observations, the relationship between the *p53* codon-72 polymorphisms and hypertrophic scar formation occurrence was investigated. Among the patients with the CCC/CCC genotype, nine patients had hypertrophic scars and 46 patients showed normal healing, which is a ratio of 0.19. However, the follow-up investigations indicated that the presence of a homozygous or heterozygous C-to-G alteration at the codon-72 site in gene *p53* resulted in 13 patients with hypertrophic scars and 192 patients with normal healing, which is a ratio of 0.07. Therefore, these results indicate that patients with the CCC/CCC genotype had a higher risk of developing hypertrophic scars compared with that for patients with the CCC/CGC or CGC/CGC genotypes.

## Introduction

Abnormal scar formation in wound healing causes aesthetic issues, particularly for female patients. Abnormal scars, including keloid and hypertrophic scars, are characterized by abnormal fibroproliferation and increased extracellular matrix production (1,2). In previous years, there have been a number of studies investigating the relationship between *p53* gene polymorphisms and breast cancer (3,4). Abnormal scars, particularly keloid scars, are considered to have specific characteristics that are similar to those of benign dermal fibroproliferative tumors. The *p53* mutations in dermal fibroblasts are considered to contribute to the formation of keloids (5,6). In addition, the relationship between *p53* polymorphisms and the susceptibility to form hypertrophic scars is an area of interest for a number of researchers (7–9). However, the majority of studies have focused on the differences between patients with abnormal scars and normal individuals. The aim of the present study was to investigate the relationship between the polymorphisms of *p53* codon-72 and the occurrence of hypertrophic scars for patients receiving a caesarean section (CS).

## Materials and methods

### Patients

**Figure 1:**
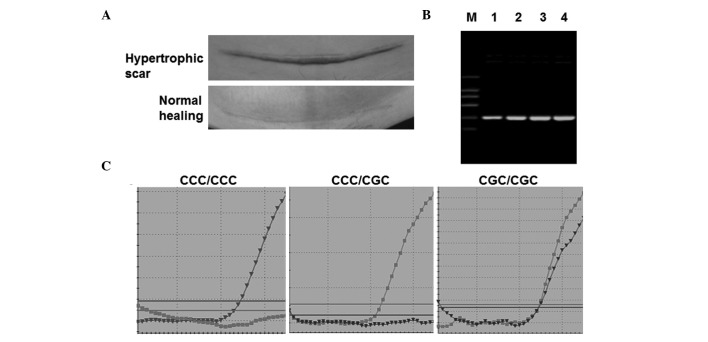
Gene *p53* codon-72 polymorphisms associated with hypertrophic scars and normal healing. (A) Representative images of a hypertrophic scar and normal healing following CS. (B) Products of nested PCR were detected following an electrophoresis reaction. A 380-bp DNA band (exon 4 of the *p53* gene) was identified in all peripheral blood samples. No non-specific amplification was observed. (C) Fluorescence PCR results of the *p53* gene codon-72 genotype. The 5′ end of CCC and CGC probes were labeled with Fam (dark gray line, ▼) and Hex (lighter gray line, ■), respectively. The 3′ end was conjugated with a quenching complex. MBC probe-positive only samples were considered as CCC/CCC homozygous, MBG probe-positive only samples were considered as CGC/CGC homozygous and MBC probe- and MBG probe-positive samples were considered to be CCC/CGC heterozygous. CS, caesarean section; Fam, carboxyfluorescein; Hex, hexachlorofluorescein.

In total, 260 female patients with an average age of 29.4 years (ranging between 20 and 39 years) and of the same ethnicity were selected from the Nanfang Hospital of Southern Medical University (Guangzhou, China) for the study. The patients did not have recorded histories of pathological scar formation or benign or malignant tumors. Blood samples were collected one week following the CS. Informed consent was obtained from each patient prior to enrollment in the study. All specimens were obtained following informed consent and procedures were conducted in accordance with the Ethical Standards of the Declaration of Helsinki (10). Written permission was provided by the hospital and the local ethics committee of Nanfang Hospital of Southern Medical University for the study. Normal healing, hypertrophic scars (Fig. 1A) and keloid scars were defined according to the following clinical features. Keloid scars became irritated two or three months following surgery and the scars were recognized as round and smooth. Keloids usually extended beyond the area of the original injury and showed no tendency to regress in the 12 months. No keloid scars were identified in the patients. Hypertrophic scars generally appeared within 4 weeks following surgery. Initially hypertrophic scar lesions were often erythematous and brownish-red but then became pale. However, hypertrophic scars never extended beyond the area of the original injury and regressed spontaneously when re-examined during the 12–18 month follow-up investigations. Normal healing scars regressed spontaneously within 3 months.

### Fluorescence quantitative polymerase chain reaction (qPCR)

Genomic DNA was extracted from 2 ml blood samples collected from the peripheral veins of each patient. A pair of molecular beacon probes were designed and labeled with fluorescent dyes to detect the single nucleotide polymorphism (SNP) of *p53* codon-72. The 5′ end of the CCC and CGC probes were labeled with carboxyfluorescein (Fam) and hexachlorofluorescein (Hex), respectively (Sangon Biotech Co., Ltd., Shanghai, China), the 3′ end was conjugated with a quenching complex [Black Hole Quencher-1 (BHQ-1); Biosearch Technologies, Inc., Novato, CA, USA]. The sequences of the probes were as follows: MBC, 5′-Fam-ATGCAGCCCCcCGTGGCCCCTGCAT-BHQ-1; and MBG, 5′-Hex-ATGCAGCCCCgCGTGGCCCCTGCAT -BHQ-1. Two primers, designed using Primer 5 software (Premier Biosoft, Palo Alto, CA, USA) and synthesized by Shanghai Genechem Co., Ltd. (Shanghai, China), were used to amplify the *p53* 380-bp fragment in exon 4. The primer sequences were as follows: Forward, TPF, 5′-GACCTGGTCCTCTGACTGCT-3′; and reverse, TPR, 5′-GATACGGCCAGGCATTGAAG-3′.

For PCR, 0.5 μl of each primer, 1 μl genomic DNA, 0.2 μl Fam-C primer and 0.2 μl Hex-G primer were mixed and then diluted with DNase- and RNase-free water to provide a total reaction volume of 20 μl. The reaction was performed with a Stratagene Mx3000P qPCR system (Agilent Technologies, Santa Clara, CA, USA). The amplified gene products were sent to Sangon Biotech Co., Ltd. (Shanghai, China) for sequencing. PCR products were detected by electrophoresis.

### Nested PCR

Nested PCR was preformed to obtain the p53 380-bp fragment in exon 4. Briefly, DNA extracted from peripheral blood was amplified by the first set of primers. The product obtained was then used as the template for a second amplification with primers of TPF and TPR. Finally, the PCR products from the second amplication were analyzed by agarose gel electrophoresis.

### Follow-up investigations

Follow-up investigations were performed for a period of 12–18 months. During this period, patients were required to return to the hospital at any time when a fibroproliferative scar appeared to grow beyond the confines of the original wound. Patients were also required to report to the hospital if symptoms, including skin erythema, pruritus or pain, developed at the site of surgical incision.

### Statistical analysis

The relationship between the *p53* gene polymorphisms and the occurrence of hypertrophic scars was evaluated by a χ^2^ test and a Student’s t-test. Moreover, the significant association between *p53* gene polymorphisms and hypertrophic scars was also evaluated by a corrected χ^2^ test. SPSS 13.0 (SPSS, Inc., Chicago, IL, USA) was used to carry out statistical analysis. P<0.05 was considered to indicate a statistically significant difference.

## Results

### Clinical characteristics of the patients

Peripheral venous blood samples were collected from 260 patients in the study. A total of 249 patients underwent the lower abdominal CS (Fig. 1A) via a transverse incision, while the remaining 11 patients underwent the traditional longitudinal CS incision.

The products of nested PCR were detected following an electrophoresis reaction. As shown in Fig. 1B, only a 380-bp DNA band was detected in all peripheral blood samples. No non-specific amplification was observed in those groups, indicating that PCR was specific.

### Fluorescence qPCR

To determine the gene *p53* codon-72 polymorphisms, fluorescence qPCR was performed. As shown in Fig. 1C, samples with only MBC probe-positive signals were considered as CCC/CCC homozygous and those with only MBG probe-positive signals were considered to be CGC/CGC homozygous. Samples with MBC and MBG probe-positive signals were considered to be CCC/CGC heterozygous. These results indicate that the three possible genotypes were successfully detected by fluorescence qPCR.

All patients received a 12–18 month follow-up investigation. During this period, hypertrophic scars developed in 22 patients, with an incidence rate of 8.47%. As shown in Table I, among the patients with the CCC/CCC genotype, nine patients had hypertrophic scars and 46 patients showed normal healing, which is a ratio of 0.19. However, the follow-up investigations indicated that the presence of a homozygous or heterozygous C-to-G alteration at the codon-72 site in gene *p53* resulted in 13 patients with hypertrophic scars and 192 patients with normal healing, which is a ratio of 0.07 (Table I). Therefore, patients with the CCC/CCC genotype had a higher risk of developing hypertrophic scars compared with that of patients with CCC/CGC or CGC/CGC genotypes. The differences between these two groups were statistically significant (P<0.05). The positive predictive value (PPV) and negative predictive value were 9/55 = 163.6/1,000 and 192/205 = 936.6/1,000, respectively. These results indicate that patients with the CCC/CCC genotype had a higher risk of developing hypertrophic scars compared with that of patients with CCC/CGC or CGC/CGC genotypes.

## Discussion

Possible mechanisms underlying the pathogenesis of hypertrophic scars include excessive inflammation, excessive angiogenesis, abnormal growth factor levels and delayed apoptosis of fibrotic myofibroblasts due to alterations to the *p53* gene (11–14). In p53-null mice, it was found that scar hypertrophy and cellular density significantly increased due to the downregulation of cellular apoptosis (15). p53 mutations in hypertrophic scars and keloid fibroblasts were detected from cultured cells (16). The *p53* codon-72 (Arg 72 Pro), which is associated with various types of cancers, is the most extensively studied SNP in the *p53* gene. Specific studies have reported that the *p53* codon-72 CGT/CCT SNP is also associated with abnormal scar susceptibility; however, this remains controversial. Wang *et al* (7) analyzed codon 72 of *p53* in 54 patients with keloids and 30 patients with hypertrophic scars using restriction fragment length polymorphism. The study observed that the frequencies of the Pro- and Arg-encoding alleles in the hypertrophic scar patients deviated significantly from those in the normal controls. However, there was no significant difference between the keloid patients and healthy individuals. Zhuo *et al* (17) analyzed 45 patients with keloids by PCR-reverse dot blotting and identified that patients with the Pro/Pro genotype had a higher risk of forming a keloid scar than patients with Pro/Arg and Arg/Arg genotypes. However, Yan *et al* (9) hypothesized that there were no significant differences in the distribution of the *p53* codon-72 polymorphism between keloid patients and healthy controls, but that the Arg/Arg genotype may affect the formation of keloids in the shoulder and back.

A study has shown that the *p53* codon-72 CCC/CCC genotype may result in keloid susceptibility in the Guangdong district (17). Furthermore, it has been reported that the Pro 72 and Arg 72 variants differ in functional activities (18). In addition, *p53* codon-72 polymorphisms may change gene expression, resulting in changes to p53 function, including activating gene transcription, inducing apoptosis and inhibiting cell transformation (7). The Pro 72 variant was shown to activate transcription effectively and upregulate the downstream genes. However, the Arg 72 variant was not only a suppressor of cellular transformation, but also induced apoptosis more effectively than the Pro 72 variant.

In the present study, the PPV of abnormal scars following CS in patients with the *p53* codon-72 CCC/CCC genotype was 163.6/1,000. Patients with the *p53* codon-72 CCC/CCC genotype were the susceptible population, whose relative risk of hypertrophic scars was 2.8896-fold higher than that of the other individuals. The results of the present study indicate that patients with the CCC/CCC genotype had a higher risk of developing hypertrophic scars compared with that of patients with CCC/CGC or CGC/CGC genotypes.

## Figures and Tables

**Table I:** Comparison between the C/C and C/G or G/G genotypes.

	*p53* polymorphisms				
				
Groups	CCC/CCC	CCC/CGC or CGC/CGC	Sum	χ^2^	P-value
Normal healing	46	192	238		
Hypertrophic scar	9	13	22		
Total	55	205	260	4.404	0.036^a^

a Compared with patients with the CCC/CCC genotype, P<0.05.
